# Transmission and Drive Involving Parasitic B Chromosomes

**DOI:** 10.3390/genes9080388

**Published:** 2018-07-31

**Authors:** R.N. Jones

**Affiliations:** Institute of Biological, Environmental and Rural Sciences (IBERS), Aberystwyth University, Edward Llwyd Building, Penglais Campus, Aberystwyth SY23 3DA, UK; neil.rnj@gmail.com

**Keywords:** B chromosomes, transmission, drive, host/parasite interaction

## Abstract

B chromosomes (Bs) are enigmatic additional elements in the genomes of thousands of species of plants, animals, and fungi. How do these non-essential, harmful, and parasitic chromosomes maintain their presence in their hosts, making demands on all the essential functions of their host genomes? The answer seems to be that they have mechanisms of drive which enable them to enhance their transmission rates by various processes of non-mendelian inheritance. It is also becoming increasingly clear that the host genomes are developing their own mechanisms to resist the impact of the harmful effects of the Bs.

## 1. Introduction

New technologies are the driver for advances in our knowledge of genetics and cytogenetics, including some fundamental questions concerning supernumerary B chromosomes (Bs): namely their origin, widespread existence, phenotypic effects, molecular organization, modes of inheritance, and population equilibrium frequencies. Their main properties can be summarised as follows: (i) they occur in thousands of species, and in all known cases they are dispensable and individuals with none are always present; (ii) they never pair with the standard A chromosomes (As) of their hosts; (iii) their behaviour at meiosis is irregular and can lead to elimination; (iv) they are usually smaller than the As and often heterochromatic; (v) several cases they have recently been shown to carry genes; (vi) they are harmful to their host organisms when present in high numbers; (vii) they have various mechanisms of accumulation, including nondisjunction and meiotic drive [1]. The context and background to the story is recorded in a number of reviews, listed in Table 1.

These reviews are augmented by the papers appearing in this special edition of Genes. The present review considers one aspect of this story, namely the modes of inheritance of Bs, with particular reference to transmission and drive which enables them to maintain their presence in populations against a gradient of harmful effects. It is convenient to deal with plants (Table 2) and animals (Table 3) separately.

## 2. Plants

There are several mechanisms of B chromosome accumulation in plants, as summarised in Table 2, of which post-meiotic nondisjunction in microspores and megaspores are the best known and are considered first. The model species, and virtually the only ones for investigating this process are rye and maize.

### 2.1. Rye (*Secale Cereal*, *2n* = 2x = 14 + Bs)

**Figure 1:**
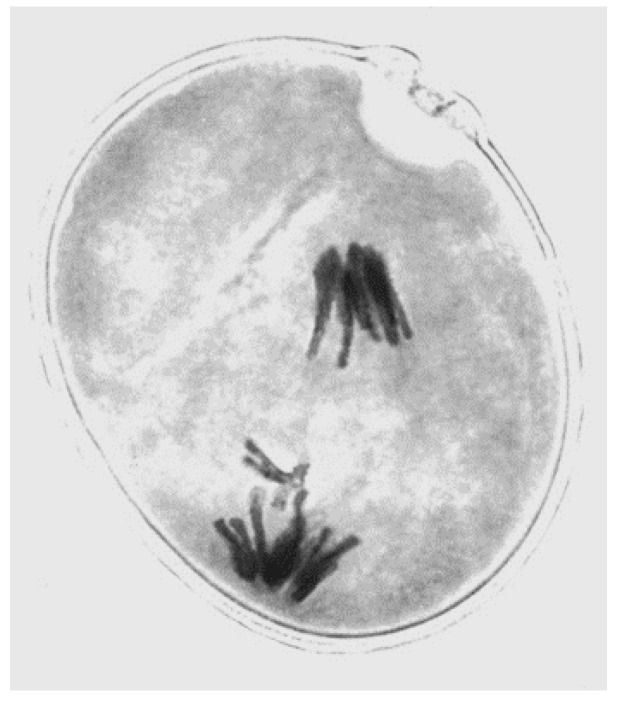
Nondisjunction of a rye B at first pollen grain mitosis (photo by author).

Directed nondisjunction at the first pollen grain mitosis in rye was discovered, described and represented diagrammatically by Hasegawa in 1934 [11]. A photograph later captured an undivided single rye B at the equator of the unequal spindle at anaphase (Figure 1).

**Figure 2:**
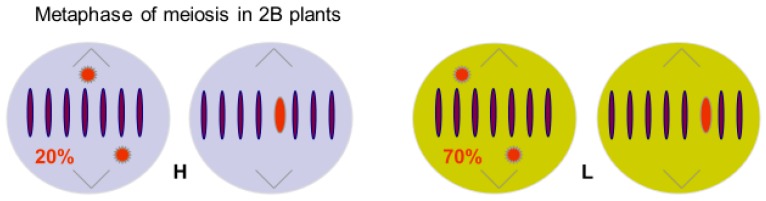
Metaphase of meiosis in 2B plants of two transmission genotypes of Korean rye, based on Jiménez et al [20]. Genotypes were selected homozygous for A chromosome ‘genes’ controlling HIGH (H) and LOW (L) transmission rates of B chromosomes. Transmission rates reflect resistance of the A background to the Bs.

Hasegawa’s diagrams are reproduced in Houben [8], together with a full account of the latest information on the cellular and molecular components of the nondisjunction process. Suffice it to say here that there is a controlling element at the end of the long arm of the B, and that a deleted B missing this region can only undergo nondisjunction when the standard B is also present and acts in trans to provide the essential function. Houben has discussed the action of this essential region in detail, together with the variation in centromere structure between that of the B and the standard A chromosomes. The sticking sites on either side of the B centromere are also considered in detail, and these comprehensive studies will not be repeated here. The autonomy of the rye B nondisjunction was established when it became known that it behaves in the same way in hexaploid wheat as it does in rye [12]; although the addition line cannot be easily maintained due to the low pairing level of the rye Bs in the wheat background [13]. This pairing observation raises the interesting point that the transmission level of the rye Bs are subject to interaction with the host background genotype, be it in a related species or in different strains of rye, and it seems that the B itself controls its own transmission properties. The output of Bs through meiosis will also clearly impact on the level at which nondisjunction can operate, determined by the number of Bs passing through to the gametophytes. Transmission data for several varieties of rye is summarized and reviewed in [14,15], and reveal a wide range of variation. Japanese populations have a B frequency of up to 90%, with most individuals having 2Bs [16], while in the Swedish variety Östgöta Gråråg it is down to as low as a few percent [17]. Müntzing also compared transmission frequencies in Östgöta Gråråg with the variety Vasa II, and found a wide variation based on meiotic pairing, but not in the nondisjunction rates. He also drew attention to the structural variation in the Bs themselves [18,19], particularly at the end of the long arm where the nondisjunction controlling element was later located. The standard B of Vasa II is larger than that of Östgöta Gråråg. Matthews and Jones [15] also concluded that the pairing rate of Bs at meiosis is the main factor determining their equilibrium frequencies in natural populations, and this pairing is a property of the Bs themselves. Jiménez et al. [20] and Puertas et al. [21], Figure 2 also report, from studies on high and low transmission lines in Korean rye, that the transmission frequency is a property of the Bs themselves, in terms of their capacity to form bivalents rather than univalents at meiosis, and does not depend directly on the level of nondisjunction in the pollen grain.

These observations lead us to consider the possibility of long-term host/parasite co-evolution, and to the idea that the parasitic effects of rye Bs might be beneficial in the long term [22]. We should also remember a classic paper by Östergren [23] on the parasitic nature of extra fragment chromosomes. This idea of the co-evolution of Bs and their hosts is a recurring theme in both plants and animals and is the basis by which the frequency of Bs in natural populations is determined.

### 2.2. Maize (*Zea Mays*, *2n* = 2x = 20 + Bs)

**Figure 3:**
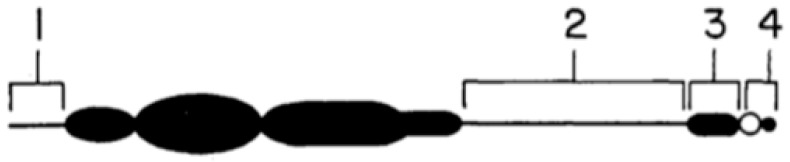
Structure of the maize B chromosome, based on Carlson [26]. See text for details of the numbering of the segments.

The mechanism of nondisjunction in maize was discovered by Roman in 1947 [24], using a translocation between the B and A chromosome 4, known as the A-B interchange TB-4a, and a marker gene to track the process. The behaviour of the B does not depend on it remaining intact. This approach was taken since the small size of the maize B in the pollen grains did not allow its behaviour to be followed cytologically. The methodology of Roman’s genetic analysis is covered in Jones and Ruban [25]. It turns out that the nondisjunction of the B occurs at the second division of the male gametophyte, which in itself does not lead to an increase in the number of Bs in subsequent generations, and it occurs in 50–100% of pollen grains [26]. The drive depends on the sperm nuclei carrying the B preferentially fertilising the egg nucleus about two thirds of the time, although how this happens is not fully understood [26]. Inheritance through the female side is normal. There are a number of studies using A-B translocations that have identified the regions of the B enable the centromere to undergo nondisjunction (Figure 3, based on Carlson [26], Jones and Ruban [25]). 

These sites, and the basis of their actions, have been extensively described [26], and are summarised briefly here. Region 1 is the short distal euchromatic tip of the B, region 2 is the longer proximal euchromatin and region 3 the proximal centromeric heterochromatin. Region 4, comprising the centromere and B short arm modifies the rate of nondisjunction. In relation to region 4, it is noteworthy that the maize B has a high level of autonomy and control over its own inheritance. This was demonstrated by Rosato et al. [27] using crosses between genotypes for high and low transmission rates in races of Pisingallo maize from Argentina. Carlson and Roseman [28] had already shown that genotypes of maize Bs could control their own transmission rates by the suppression of meiotic loss. Drive mechanisms based on pollen grain mitosis are well known in several other species, many of which listed in Table 2 with references. There are other mechanisms of drive that are not based on pollen grain mitosis (Table 2), and some of these are worthier of more detailed description.

Kimura and Kayano [33] were first to describe a mathematical theory to analyse the mechanism of distribution of Bs in a natural plant population. Their theory also suggested a mechanism by which the deleterious effect of Bs could be reduced in the course of evolution. Female meiotic drive is also noted in three other cases (Table 2). In *Crepis capillaris*, there is somatic nondisjunction coincident with flower initiation [40]. It is noteworthy that there is a number species with widespread B polymorphisms where no mechanism of drive could be determined (*Allium schoenoprasum*, *Xanthisma texanum*, *Centauria scabiosa)*, and numerous others where we have no specific information, or species (most of them) where transmission has not yet been determined. There is a very narrow basis on which our current view of drive and B/A co-evolution is built.

In *Lilium callosum* the transmission of Bs through the pollen is normal, but there is drive through the female line which occurs at meiosis [33]. The spindle is asymmetrical at metaphase I and single unpaired Bs tend to be located 80% of the time in the larger area of the spindle which will give rise to the egg cell, and only 20% of the time in the area which will give rise to the embryo. An interesting feature of this process is that it depends on an asymmetric spindle, like the pollen mitosis in rye and other Gramineae.

## 3. Animals

**Figure 4:**
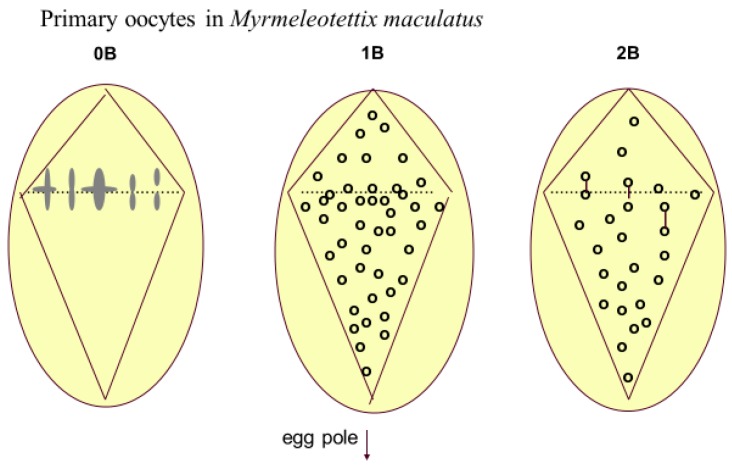
Meiotic drive in primary oocytes of *Myrmeleotettix maculatus*, based on Hewitt [48]. The 0B nucleus shows the seven A chromosome bivalents lined up at the equator of the asymmetrical spindle at metaphase I. In the 1B and 2B nuclei there are more Bs in the region of the nucleus which will form the egg cells. 2B bivalents are shown as joined dots. This preferential chromosome distribution matches very closely the level of preferential transmission determined from breeding experiments.

In most of the animal kingdom gametes are produced directly by meiosis. There is no gametophyte phase to the life cycle and female or male drive is mainly based on B-behaviour at meiosis. *Eyprepocnemis plorans* [43] is the most intensively studied, and best understood in terms of population dynamics, of the species lacking drive. The authors explain that in a newly invaded population, a particular form of the B has substantial drive, and that co-evolution of drive between the nascent B and suppressor genes in the As gradually reduces the level of B-drive. The B eventually becomes neutral and stochastic loss then eliminates it from the population. When a new variant of the B appears with drive it can displace the original variant, and a new cycle of drive suppression and drift to extinction occurs. The time it takes for extinction to occur is related to population size [58]. In *Eyprepocnemis plorans* [43] the transmission pattern of three variants of the Bs was studied in detail at meiosis in both male and female animals and in controlled crosses. The Bs appear to be inherited in a regular manner with no tendency to either accumulation or loss, which begs the question of their status in the population. The idea that they are neutral, based on some historic perspective, or that they have an accumulation mechanism unrelated to meiotic drive (mating preferences or sperm precedence), or natural selection can all be advanced, but at the end of the day there is no definitive evidence to answer the questions. No net accumulations of Bs was found in the fish species *Prochilodus lineatus* [44], and the argument was again advanced that the Bs were possibly driven in the past but this was neutralised by drive-suppressor genes in the A genome. Nur and Brett [47] reported on genotypes suppressing meiotic drive in *Pseudococcus obscurus*. Female meiotic drive is the most common mode of transmission and is best known in grasshoppers and in the mealy bug *Pseudococcus obscuruus.* Preferential segregation of univalent Bs at meiosis is one of the mechanisms of B-drive in animals, and this has usually been inferred from breeding experiments, as in *Melanoplus femur-rubrum* [49], although there is only one case in which this has been demonstrated at cytological level and that is in *Myrmeleotettix maculatus*, Figure 4 [48].

Mechanisms of male drive and of male drive and female drag have also been reported (Table 2), as well as specialised systems -in *s*, *Eumigus monticola*, and *Eyprepocnemis plorans*. In the wasp *Nasonia vitripennis* the males are haploid and develop from unfertilized eggs, while the diploid females develop from fertilized eggs. Some individuals in this species carry a genetic element, termed PSR (paternal sex ratio). This element is transmitted through sperm where it causes condensation and subsequent loss of paternal chromosome set in fertilized eggs. Diploid females are thereby converted into haploid males. The authors have shown that PSR has the properties of a supernumerary chromosome B with a number of B-specific repetitive sequences. The PSR appears to produce a trans-acting product which causes condensation of the paternal chromosome set, but is not itself affected. The fascinating effect is that the PSR enhances its own transmission by eliminating the rest of the genome, and can thus considered to be the ultimate ‘selfish’ genetic element.

## 4. Conclusions

Among the relatively small number of species that have been investigated, relative to the total number of known species, there are a variety of mechanisms of B chromosome drive, including those where no drive has been identified. In plants, the most common process takes place post-meiotically whereas in animals it mainly involves meiosis. In several cases of both plants and animals, the population equilibrium B frequency is a result of conflict between the As and Bs: Bs providing the drive and As acting in various ways to suppress the increase in B numbers. Even within populations there are differences in B equilibrium frequencies between different populations. The most instructive case is that of the grasshopper *Eyprepocnemis plorans*, where suppressor genes in the As have neutralised one of the B-types, and over time stochastic events can eliminate that B from the population. Eventually, a new B can arise with its drive restored and the cycle can be repeated over again. In the plant world, there is also variation in B transmission in certain populations, particularly in maize and in rye, and evidence of suppression of B-drive by the A genomes. However, there are no cases known as yet where the As have completely suppressed and neutralised any B. The view that Bs are nuclear parasites, albeit involved in host/parasite interactions, seems to hold true with the balance in favour of the parasites rather than the host. There is much more to learn about this aspect of the story of Bs, involving the species which have been investigated as well as the many others about which we have no knowledge.

## Figures and Tables

**Table 1:** Selection of reviews on B chromosomes.

Author	Title	Reference
Jones, R.N.	B Chromosome Drive	[1]
Jones, R.N. et al.	B Chromosomes	[2]
Camacho, J.P.M.	B Chromosomes	[3]
Houben, A.; et al.	Biology and Evolution of B Chromosomes	[4]
Houben, A.; et al.	Evolution and Biology of Supernumerary B Chromosomes	[5]
Banaei-Moghaddam, A.M. et al.	Genes on B Chromosomes: Old Questions Revisited with New Tools	[6]
Valente, G.T. et al.	B Chromosomes: from Cytogenetics to Systems Biology	[7]
Houben, A.	B Chromosomes—A Matter of Chromosome Drive	[8]
Ruban, A. et al.	How Next-Generation Sequencing Has Aided Our Understanding of the Sequence Composition and Origin of B Chromosomes	[9]
Coan, R.L.B. et al.	Landscape of Transposable Elements Focusing on the B Chromosome of the Cichlid Fish *Astatotilapia latifasciata*	[10]

**Table 2:** Summary of various mechanisms of B accumulation in plants.

**Nondisjunction at First Pollen Grain Mitosis**
*Aegilops speltoides*, *Alopecurus pratensis*, *Anthoxanthum aristatum*, *Brachycome lineariloba*, *Briza media. Dactylis glomerata*, *Deschampsia bottnica*, *Deschampsia caespitosa*, *Deschampsia wibeliana*, *Festuca arundinacea*, *Festuca pratensis*, *Haplopappus gracilis*, *Holcus lanatus*, *Phleum phleoides*, [2] *Panicum maximum* [29] *Aegilops mutica* [30]
**Pollen Grain Mitosis of Extra Divisions**
*Sorghum-purpureo-sericium* [31]
**Somatic Non-Disjunction in the Developing Inflorescences**
*Crepis capillaris* [32]
**Female Meiotic Drive**
*Lilium callosum* [33], *Phleum nodosum* [34] *Plantago serraria* [35] *Trillium grandiflorum* [36]
**Female Meiotic Drive and Male Meiotic Drag**
*Picea sitchensis* [37] *Hypochoeris maculata* [38]
**Male Drive**
*Haplopappus validus*, *Clarkia elegans*, *Iseilema laxum* [2] *Briza humilis* B^L^ [39]
**Somatic Nondisjunction Coincident with Flower Initiation**
*Crepis capillaris* [40]
**No Apparent Mechanism**
*Allium schoenoprasum* [41] *Xanthisma texanum* [42] *Centauria scabiosa*, *Poa alpina*, *Ranunculus acris*, *Ranunculus ficaria* [2].

**Table 3:** Summary of various mechanisms of B-chromosome accumulation in animals.

**No Drive**
*Eyprepocnemis plorans* (grasshopper) [43] *Prochilodus lineatus* (fish) [44] *Metagagrella tenuipes* (Arachnida, Japanese harvestman) [45]
**Mechanism (?) to Boost B-number in Males**
*Euphydryas colon* (Lepidoptera)*.* [46]
**Female Meiotic Drive**
*Pseudococcus obscurus* (mealy bug) [47] *Myrmeleotettix maculatus* (grasshopper) [48] *Melanoplus femur-rubrum* (grasshopper) [49] *Heteracris littoralis* (grasshopper) [50] *Omocestus burri* (grasshopper) [51]
**Male Drive**
*Rattus fuscipes* (Australian bushrat) [52]
**Male Meiotic Drive and the Opposite of Drive, i.e., Female Drag**
*Chortoicetes terminifera* (Australian plague locust) [53]
**PSR Enhances Transmission by Losing Paternal Chromosomes Except Itself**
*Nasonia vitripennis* (parasitic wasp) [54]
**Female Meiotic Drive and Male Meiotic Drag**
*Myrmeleotettix maculatus* (grasshopper) [55] *Locusta migratoria* [56]
**B Elimination during Spermiogenesis**
*Eumigus monticola*, *Eyprepocnemis plorans* [57]
